# Biosynthesis of Akaeolide and Lorneic Acids and Annotation of Type I Polyketide Synthase Gene Clusters in the Genome of *Streptomyces* sp. NPS554

**DOI:** 10.3390/md13010581

**Published:** 2015-01-16

**Authors:** Tao Zhou, Hisayuki Komaki, Natsuko Ichikawa, Akira Hosoyama, Seizo Sato, Yasuhiro Igarashi

**Affiliations:** 1 Biotechnology Research Center and Department of Biotechnology, Toyama Prefectural University, 5180 Kurokawa, Imizu, Toyama 939-0398, Japan; E-Mail: t276001@st.pu-toyama.ac.jp; 2 Biological Resource Center, National Institute of Technology and Evaluation (NBRC), 2-5-8 Kazusa-kamatari, Kisarazu, Chiba 292-0818, Japan; E-Mail: komaki-hisayuki@nite.go.jp; 3 Biological Resource Center, National Institute of Technology and Evaluation (NBRC), 2-4-19 Nishihara, Shibuya-ku, Tokyo 151-0066, Japan; E-Mails: ichikawa-natsuko@nite.go.jp (N.I); hosoyama-akira@nite.go.jp (A.H.); 4 Central Research Laboratory, Nippon Suisan Kaisha, Ltd., Tokyo Innovation Center, 1-32-3 Nanakuni, Hachioji, Tokyo 192-0991, Japan; E-Mail: s-satou@nissui.co.jp

**Keywords:** polyketide, marine actinomycete, *Streptomyces*, biosynthesis, genome

## Abstract

The incorporation pattern of biosynthetic precursors into two structurally unique polyketides, akaeolide and lorneic acid A, was elucidated by feeding experiments with ^13^C-labeled precursors. In addition, the draft genome sequence of the producer, *Streptomyces* sp. NPS554, was performed and the biosynthetic gene clusters for these polyketides were identified. The putative gene clusters contain all the polyketide synthase (PKS) domains necessary for assembly of the carbon skeletons. Combined with the ^13^C-labeling results, gene function prediction enabled us to propose biosynthetic pathways involving unusual carbon-carbon bond formation reactions. Genome analysis also indicated the presence of at least ten orphan type I PKS gene clusters that might be responsible for the production of new polyketides.

## 1. Introduction

Natural products are the essential source of small molecules with various biological activities. The urgent need for new drugs in response to drug-resistance development among pathogens and increasing incidence rates for cancer have been impelling the discovery of new sources and development of methodology in exploring novel chemical entities [1]. Because of the declining efficiency in terrestrial microbial metabolite research in recent years, marine research is attracting more attention for a vast reservoir of biological and chemical diversity than ever [2]. In particular, marine *Streptomyces*, widely spread in marine environments, has been recognized as an emerging source of new bioactive compounds [3].

Microbial secondary metabolites are classified largely into several structural types, polyketides, peptides, terpenoids, aminoglycosides, and alkaloids, according to the biosynthetic pathway [4]. Of these, polyketides are the most structurally diverse metabolites commonly produced by actinomycetes. The carbon skeleton of polyketides is constructed from small building blocks along the assembly line, known as polyketide synthase (PKS). Aliphatic polyketides, often associated with numerous substituents and chiral centers, are assembled by modular-type PKSs. This multifunctional enzyme contains catalytic domains necessary for chain elongation with distinct conserved amino acid sequences, which allows prediction of chain length, oxidation level of carbons, and thus the approximate overall structure of final products through annotating the gene functions [5]. Owing to the accumulation of microbial genomic data in public databases, genome mining has become a powerful tool to screen candidate organisms capable of producing novel metabolites, using specific DNA sequences of interest [6,7].

**Figure 1:**
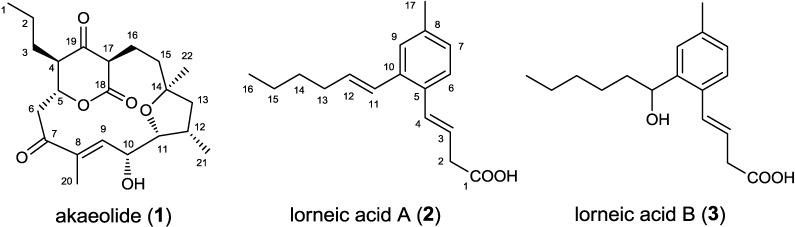
Structures of akaeolide (**1**) and lorneic acids A (**2**) and B (**3**).

In our previous studies, two new unique polyketide classes, akaeolide (**1**) and lorneic acids A (**2**) and B (**3**) were isolated from *Streptomyces* sp. NPS554 collected from marine sediment near the coast of Miyazaki, Japan (Figure 1). Akaeolide (**1**) has an unusual 15-membered carbocyclic framework containing a β-keto-δ-lactone unit [8]. This compound displays moderate antimicrobial activity against *Micrococcus luteus* with an MIC value of 25 μg/mL (64 μM) and cytotoxicity to 3Y1 rat fibroblasts with an IC_50_ value of 8.5 μM. Lorneic acids have a fatty acid-like structure in which a benzene ring is embedded. They inhibit phosphodiesterases with selectivity toward PDE5 with IC_50_ values of submicromolar range [9]. Despite the structural rarity of these compounds, biosynthetic origins remain unknown. We herein report the biosynthesis of akaeolide and lorneic acid elucidated by isotope precursor feeding and by annotation of biosynthetic genes. Annotation of unknown type I PKS gene clusters responsible for the production of new polyketides is also presented.

## 2. Results and Discussion

### 2.1. Incorporation of ^13^C-Labeled Precursors

**Figure 2:**
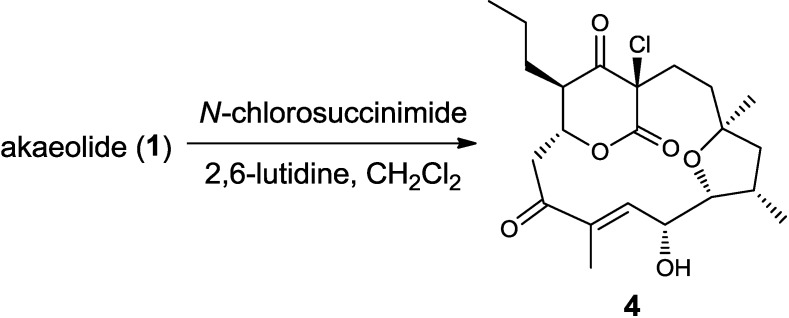
Conversion of akaeolide (**1**) to 17-chloroakaeolide (**4**).

Inspection of the carbon connectivity and the position of carbon branches suggested that **1** and **2** were synthesized through the polyketide pathway. In order to elucidate the biosynthetic origin and incorporation pattern, strain NPS554 was cultured in the presence of plausible biosynthetic precursors labeled with carbon-13, namely [1-^13^C]acetate, [2-^13^C]acetate, and [1-^13^C]propionate, which could be incorporated into the polyketide backbone via acyl CoA carboxylation. According to our previous study, **1** exists as a mixture of several tautomeric isomers in NMR solvents caused by the enolization at C-17, consequently giving multiple ^13^C signals for each carbon [8]. This was undesirable to the quantification of carbon intensity; therefore, the purified ^13^C-labeled **1** was converted to the chlorinated derivative **4** which could not undergo isomerization (Figure 2).

**Figure 3:**
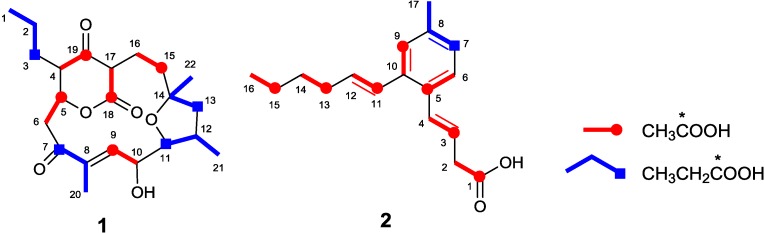
Incorporation of ^13^C-labeled precursors into akaeolide (**1**) and lorneic acid A (**2**).

The relative enrichment of each carbon by the incorporation of ^13^C-labeled precursors was determined by ^13^C NMR measurement (Table 1). Enrichments at C-5, C-9, C-15, C-18, and C-19 were observed by the feeding of [1-^13^C]acetate while C-3, C-7, C-11, and C-13 were highly enriched by [1-^13^C]propionate feeding (Table 1, Supplementary Figures S1 and S2). C-12 and C-15 were overlapped at 34.7 ppm in the ^13^C NMR spectrum, but the signal enhancement was ascribed to the incorporation of [1-^13^C]acetate into C-15 because the three-carbon fragment C-21/C-12/C-11 was derived from a propionate as proven by [1-^13^C]propionate incorporation into C-11 (Figure 3).

C-16 and C-17 were labeled by [2-^13^C]acetate and C-15 and C-18 by [1-^13^C]acetate (Figure 3). This labeling pattern is inconsistent with normal polyketide chain elongation but could be explained by incorporation of succinate, which can be labeled by ^13^C-labeled acetates in TCA cycle [10,11]. However, the feeding of 1,4-^13^C_2_-succinic acid gave no signal enhancement for C-15 and C-18 of akaeolide (Supplementary Figure S2). Therefore, C-16 and C-17 should be connected after the polyketide chain assembly.

**Table 1:** Incorporation of ^13^C-labeled precursors into 17-chloroakaeolide (**4**) and lorneic acid A (**2**).

Position	17-chloroakaeolide (4)				Lorneic Acid A (2)		
	Relative Enrichments ^α^						
	δ_C_	[1-^13^C]acetate	[2-^13^C]acetate	[1-^13^C]propionate	δ_C_	[1-^13^C]acetate	[1-^13^C]propionate
1	14.5	1.01	0.88	0.83	177.7	**7.25**	0.88
2	19.0	1.00	1.00	1.00	38.3	1.04	1.02
3	28.8	1.00	1.69	**32.90**	121.8	**6.71**	1.03
4	46.8	0.99	**4.44**	0.99	131.9	0.99	1.01
5	75.4	**5.01**	1.06	0.81	131.7	**6.53**	0.83
6	43.5	1.14	**4.83**	0.90	126.4	0.97	1.01
7	203.1	1.17	2.11	**40.33**	127.8	1.03	**42.61**
8	138.9	0.94	0.77	0.58	137.3	0.93	0.73
9	140.6	**4.60**	1.02	0.84	127.0	**6.49**	1.13
10	69.0	1.04	**4.47**	0.61	136.1	0.80	0.81
11	84.1	0.96	1.60	**30.19**	127.4	**6.13**	1.13
12	34.7	**4.88**	1.03	0.87	133.7	0.90	1.00
13	49.1	0.99	1.61	**31.63**	33.0	**6.35**	1.07
14	83.1	0.99	0.90	0.94	31.6	1.02	1.00
15	34.7	1.00	1.03	0.87	22.3	**6.32**	1.04
16	30.1	1.00	**4.33**	1.02	13.9	1.00	1.00
17	63.7	0.96	**3.77**	1.12	21.2	1.01	0.99
18	164.5	**4.79**	0.94	0.79			
19	196.4	**4.41**	1.02	1.07			
20	14.5	1.01	0.88	0.83			
21	25.1	1.00	0.82	0.99			
22	14.6	1.01	0.88	0.83			

^α^ ^13^C signal intensity of each peak in the labeled compounds was divided by that of the unlabeled carbon, normalized to give an enrichment ratio. C-2 was chosen as an unlabeled carbon for 17-chloroakaeolide and C-16 for lorneic acids. The numbers in bold type indicate ^13^C-enriched atoms from ^13^C-labeled precursors.

Biosynthetic origin of lorneic acid A (**2**) was similarly elucidated by feeding experiments with [1-^13^C]acetate and [1-^13^C]propionate. The relative enrichment of each carbon by the incorporation of ^13^C-labeled precursors is summarized in Table 1. Enrichments at C-1, C-3, C-5, C-9, C-11, C-13, and C-15 were observed with the incorporation of [1-^13^C]acetate, while only C-7 was labeled by [1-^13^C]propionate feeding (Supplementary Figure S3). This labeling pattern obviously indicates that the carbon chain extension begins with C-16/C-15 acetate, followed by the condensation of three malonates, one methylmalonate, and three malonates, and terminates at C-1 (Figure 3).

### 2.2. Genome Analysis and Annotation of Biosynthetic Genes

Akaeolide (**1**) and lorneic acids (**2** and **3**) are synthesized through the polyketide pathway. Our next attention was focused on how the extra C–C bond is formed in akaeolide biosynthesis and how the benzene ring of lorneic acids is constructed from a linear precursor. To get insight into more detailed biosynthetic mechanism, whole genome shotgun sequencing was carried out for *Streptomyces* sp. NPS554 to identify the biosynthetic gene clusters. At least twelve modular type I polyketide synthase (type I PKS) gene clusters are present in the genome (Supplementary Table S1). Eight clusters from #1 to #8 were completely sequenced but the remaining four clusters, from #9 to #12 were partial, likely divided into two or more scaffolds in the draft genome sequences.

Since the polyketide backbones of akaeolide and lorneic acids are constructed from one acetate starter unit and seven extender units, each cluster should have eight PKS modules. Among the PKS gene clusters present in this strain, clusters #1 and #6 are comprised of one loading module and seven extension modules, totally eight PKS modules. On the basis of predicted substrates for acyltransferase (AT) domains and the presence or absence of ketoreductase (KR), dehydratase (DH), and enoylreductase (ER) domains, clusters #1 and #6 could be assigned to lorneic acid and akaeolide biosynthetic clusters, respectively.

### 2.3. Biosynthetic Gene Cluster for Akaeolide

**Figure 4:**
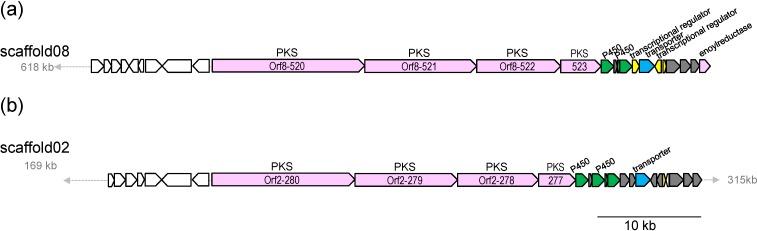
Gene organization of the akaeolide biosynthetic gene cluster of *Streptomyces* sp. NPS554 (**a**) and its orthologous gene cluster in *Lechevalieria aerocolonigenes* NBRC 13195^T^ (**b**).

**Figure 5:**
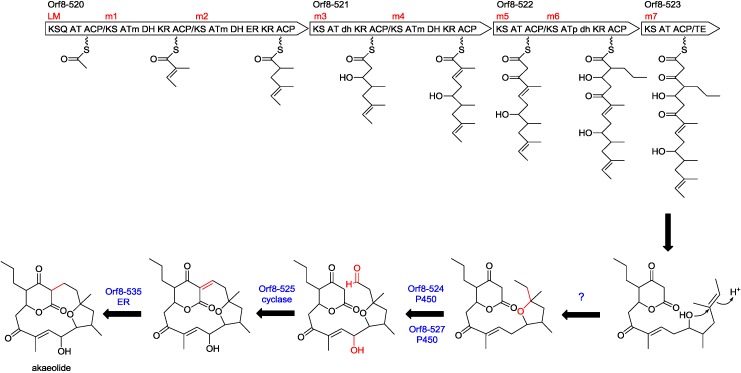
PKS domain organization and plausible biosynthetic pathway for akaeolide.

Gene organization of the biosynthetic gene cluster for akaeolide (**1**) and proposed functions of the PKS genes and the neighboring genes are summarized in Table 2. This gene cluster consists of four PKS genes with eight modules (Figure 4a). AT domains in loading module (LM), module 3 (m3), m5, and m7 have signature amino-acid residues specific to malonyl-CoA (C_2_), while those of m1, m2, and m4 have that for methylmalonyl-CoA (C_3_) [12,13]. The signature amino-acid residue of m6 predicted its substrate as an alkylmalonyl-CoA, specifically propylmalonyl-CoA (C_5_) in this case as suggested by the structure of the product [14,15,16]. These annotation results indicate that the PKSs assemble the polyketide carbon chain by sequential incorporation of C_2_, C_3_, C_3_, C_2_, C_3_, C_2_, C_5_, and C_2_ units. In combination with the results from ^13^C-labeling experiments, we could propose that the chain extension starts from C-16/C-15 acetate unit, followed by a sequence of condensation with two methylmalonates, one malonate, one methylmalonate, one malonate, and one propylmalonate, and terminates at C-18 by incorporation of one malonate (Figure 3). In multimodular type I PKS pathways, combination of DH, ER, and KR domains in each module regulates the oxidation level of carbons in the polyketide chain [5]. This PKS cluster has four DH/KR domains and one DH/ER/KR domain, corresponding to four double bonds and one saturated methylene formation, respectively, but the actual product has only two double bonds. DH domains in m3 and m6 are likely not functional (represented by “dh” in Figure 5). A quite similar gene cluster is present in the genome of *Lechevalieria aerocolonigenes* NBRC 13195^T^, which will be discussed later (Figure 4b).

**Table 2:** Proposed functions of type I PKS genes for akaeolide biosynthesis and neighboring genes.

Orf8-	Size ^a^	Proposed Function	Protein Homolog, *Origin*, Accession Number	% ^b^
511	413	ligase	ligase, *Streptomyces* sp. NRRL S-118, WP_031071281	86/92
512	242	glutamine amidotransferase	glutamine amidotransferase, *Streptomycetaceae* bacterium MP113-05, EST22401	86/91
513	315	unknown	membrane protein, *S. hygroscopicus*, WP_030828871	76/83
514	248	type II thioesterase	thioesterase, *S. mobaraensis*, EME98724	64/76
515 ^c^	294	methyltransferase	methyltransferase type 12, *S. bingchenggensis*, ADI12483	70/82
516 ^c^	180	unknown	GCN5 family acetyltransferase, *Streptomyces* sp. NRRL S-87, WP_030200822	68/78
517	532	acyl CoA carboxylase α-subunit	carboxyl transferase, *S. rimosus*, ELQ79111	82/89
518 ^c^	947	transcriptional regulator	hypothetical protein, *Lechevalieria aerocolonigenes*, WP_030473578	45/57
519 ^c^	532	oxidoreductase	hypothetical protein, *L. aerocolonigenes*, WP_030473579	64/76
520	4917	type I PKS	putative type I polyketide synthase, *S. bingchenggensis*, ADI04502	53/64
521	3601	type I PKS	polyketide synthase, *S. rapamycinicus*, AGP57755	53/64
522	2771	type I PKS	type I polyketide synthase, *S. flaveolus*, ACY06287	52/63
523	1322	type I PKS	hypothetical protein, partial, *S. novaecaesareae*, WP_033330653	52/61
524	408	cytochrome P450	cytochrome P450, *L. aerocolonigenes*, WP_030470230	72/84
525	115	cyclase	hypothetical protein, *L. aerocolonigenes*, WP_030470229	63/79
526	78	ferredoxin	hypothetical protein BN6_14320, *Saccharothrix espanaensis* DSM 44229, CCH28755	61/73
527	402	cytochrome P450	cytochrome P450, *L. aerocolonigenes*, WP_030470226	73/84
528	229	transcriptional regulator	NmrA family transcriptional regulator, *L. aerocolonigenes*, WP_030470224	76/84
529	498	transporter	Puromycin resistance protein pur8, partial, *L. aerocolonigenes*, WP_030470223	66/76
530^c^	216	transcriptional regulator	NmrA family transcriptional regulator, *L. aerocolonigenes*, WP_030470222	70/82
531	109	transcriptional regulator	HxlR family transcriptional regulator, *L. aerocolonigenes*, WP_030470221	69/83
532	457	enoyl-CoA reductase/carboxylase	NADPH:quinone reductase, *L. aerocolonigenes*, WP_030470219	79/87
533	340	3-oxoacyl ACP synthase	3-oxoacyl-ACP synthase, *S. olivaceus*, WP_031035846	70/82
534	286	3-hydroxyacyl CoA dehydrogenase	3-hydroxybutyryl-CoA dehydrogenase, *L. aerocolonigenes*, WP_030470217	63/80
535	339	enoylreductase	putative succinate-semialdehyde dehydrogenase (acetylating), *S. afghaniensis*, EPJ38274	68/77
536	>213 ^d^	transposase	transposase, *Streptomyces* sp. NRRL B-24484, WP_030264850	78/87

^a^ in aa; ^b^ identity/similarity; ^c^ encoded in complementary strand; ^d^ located at scaffold terminal.

Based on the results obtained from gene analysis (Table 2), biosynthetic pathway for akaeolide is proposed (Figure 5). First, the polyketide backbone is synthesized by four type I PKSs, Orf8-520 to -523 containing all the catalytic domains necessary for the construction of the carbon framework. According to the KR fingerprints rule, both of the KR domains in modules 2 and 6 belong to B1-type, suggesting that the absolute configuration of hydroxyl groups at C-5 and C-11 is “*S*” and that of the propyl group at C-4 is “*R*” [17]. The absolute configuration of the methyl group at C-12 was suggested to be “*S*” on the basis of amino acid alignment in the ER domain of module 2 [18]. These predictions were completely consistent with the stereochemistry of the actual natural product. After the release from the enzyme along with lactone formation, the putative linear product might undergo cyclic ether formation although it was unable to assign any genes responsible for this cyclization reaction only from the annotation data. Two cytochromes P450 would be involved in hydroxylation at C-10 and oxidation of the methyl group at C-16 to the aldehyde. The next step could be carbon-carbon bond formation between the activated methylene C-17 and the aldehyde at C-16. This reaction would be catalyzed by a putative cyclase Orf8-525 that was predicted to have a similar function to SnoaL on the basis of domain homology by InterPro [19]. SnoaL is a polyketide cyclase that catalyzes aldol condensation in the biosynthesis of nogalamycin, an aromatic polyketide produced by *Streptomyces* [20,21]. After dehydration from the aldol product, Orf8-535 coding enoylreductase would act to reduce the conjugated C–C double bond between C-16 and C-17. These functional assignments are mostly reasonable to explain the biosynthesis of akaeolide (**1**).

### 2.4. Biosynthetic Gene Cluster for Lorneic Acid

**Figure 6:**
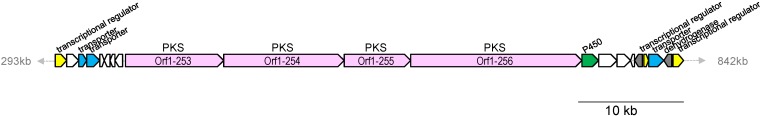
Gene organization of the biosynthetic gene cluster for lorneic acids.

**Figure 7:**
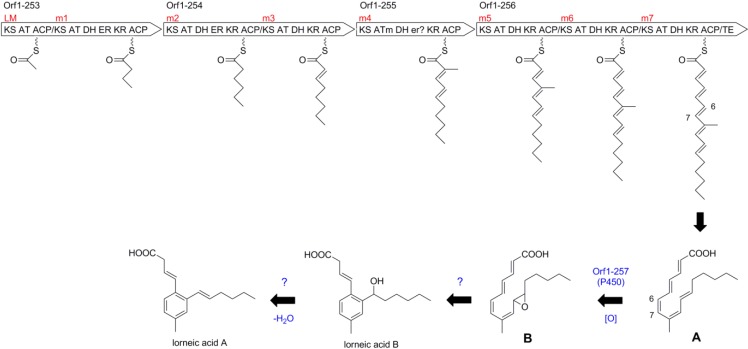
Proposed biosynthetic pathway for lorneic acids.

Gene organization of the biosynthetic gene cluster for lorneic acids (**2** and **3**) is shown in Figure 6 and proposed functions of the PKS genes and the neighboring genes are summarized in Table 3. The PKS gene cluster is composed of one loading module and seven extension modules (Figure 7). Signature amino-acid residues of AT domains [12,13] predicted the substrate specificity: acetyl-CoA (C_2_) would be loaded onto LM, malonyl-CoA (C_2_) onto m1, m2, m3, m5, m6, and m7, and methylmalonyl-CoA (C_3_) onto m4. Therefore, the polyketide carbon chain would be assembled by sequential incorporation of C_2_, C_2_, C_2_, C_2_, C_3_, C_2_, C_2_, and C_2_ units, consistent with the results obtained from the ^13^C-labeling experiments (Figure 3). The PKS cluster contains three DH/ER/KR domains in m1, m2, and m4 and four DH/KR domains in m3, m5, m6, and m7, corresponding to three saturated methylene carbons and four double bonds, respectively. However, the ER domain in m4 might be unfunctional if a putative intermediate **A** (Figure 7) were generated as described below [22].

**Table 3:** Proposed functions of type I PKS genes for lorneic acid biosynthesis and neighboring genes.

Orf1-	Size ^a^	Proposed Function	Protein Homolog, *Origin*, Accession Number	% ^b^
245	372	transcriptional regulator	transcriptional regulator, *Cellulosimicrobium cellulans*, WP_024839151	80/89
246	376	unknown	hypothetical protein, *C. cellulans*, WP_024839150	76/84
247	255	ABC transporter	sugar ABC transporter ATP-binding protein, *C. cellulans*, WP_024839149	79/86
248	418	ABC transporter	ABC transporter permease, *C. cellulans*, WP_024839148	74/82
249	138	unknown	hypothetical protein AOR_1_82014, *Aspergillus oryzae*, XP_003189833	50/60
250 ^c^	185	unknown	hypothetical protein, *Streptomyces* sp. CNQ766, WP_018834862	24/43
251 ^c^	158	unknown	hypothetical protein, *Jiangella gansuensis*, WP_026874523	56/66
252 ^c^	239	unknown	hypothetical protein, *Actinopolymorpha alba*, WP_020576671	60/70
253	3128	type I PKS	hypothetical protein, *Nocardia* sp. BMG51109, WP_024802962	59/70
254	3893	type I PKS	hypothetical protein, *Nocardia* sp. BMG51109, WP_024802962	58/69
255	2176	type I PKS	hypothetical protein, *Nocardia* sp. BMG51109, WP_024802960	68/78
256	5477	type I PKS	hypothetical protein, *Nocardia* sp. BMG51109, WP_024802963	62/72
257	534	cytochrome P450	cytochrome P450, *Micromonospora* sp. ATCC 39149, EEP70569	73/84
258	588	unknown	hypothetical protein (glycosidase), *Streptomyces* sp. CNY243, WP_018851787	79/87
259	435	unknown	beta-lactamase, *Catenulispora acidiphila*, ACU74739	80/87
260 ^c^	118	unknown	PDZ and LIM domain protein 1, partial, *Columba livia*, EMC79925	31/52
261 ^c^	254	methyltransferase	methyltransferase, *S. avermitilis*, BAC68374	84/89
262	148	transcriptional regulator	Rrf2 family transcriptional regulator, *Nocardia* sp. BMG51109, WP_024804268	78/86
263	486	transporter	MFS transporter, *Nocardia* sp. BMG51109, WP_024804269	68/80
264 ^c^	250	short-chain dehydrogenase	short-chain dehydrogenase, *A. alba*, WP_020577481	92/94
265	331	transcriptional regulator	AraC family transcriptional regulator, *A. alba*, WP_020577482	92/94

^a^ in aa; ^b^ identity/similarity; ^c^ encoded in complementary strand.

Annotation of the domain function for the PKS genes indicated the formation of a linear polyolefinic acid starting from an acetate to which six malonates and one methylmalonate are condensed (Figure 7). This polyene intermediate tethered to the PKS would have all-*trans* double bonds because the KR domain in module 5 is B-type [23]. After being released from the enzyme, the intermediate would undergo *cis*/*trans*-double bond isomerization at C-6/C-7 and cyclization. Orf1-257 located downstream of the PKS genes shows high homology to cytochromes P450, suggesting the involvement of P450-mediated monooxygenation in the aromatization of the polyolefinic precursor (**A**) and the introduction of a hydroxyl group at C-11. Epoxidation of **A** by Orf1-257 would give an epoxide (**B**), which could undergo aromatization through an unknown pathway to provide lorneic acid B (**3**) (Figure 7). Further dehydration might afford lorneic acid A (**2**). This pathway is likely plausible since the addition of one oxygen atom to the intermediate **A** (C_17_H_24_O_2_ + O) can give rise to the intermediate **B** that has the same molecular formula as that of lorneic acid B (**3**, C_17_H_24_O_3_).

### 2.5. Distribution of Biosynthetic Gene Clusters for Akaeolide and Lorneic Acid in Other Strains

Akaeolide (**1**) and lorneic acids (**2** and **3**) have uncommon structural features, prompting us to investigate the distribution of related biosynthetic genes in other organisms. Homologues of PKS genes for akaeolide and lorneic acid biosynthesis were searched in GenBank DNA database by using BLAST [24]. An orthologous gene cluster to akaeolide cluster was likely present in *L. aerocolonigenes* NRRL B-3298^T^ (Table 2) but its presence was unclear because the cluster was divided into several contigs (contig25.1, contig88.1, contig59.1 *etc*.) and not completely sequenced. Therefore, we undertook genome sequencing of *L. aerocolonigenes* NBRC 13195^T^ (the same strain as NRRL B-3298^T^, deposited at NBRC, Japan) to obtain the complete gene sequence. PKS domain organization of the akaeolide cluster ortholog in *L. aerocolonigenes* NBRC 13195^T^ was in good agreement with that of the akaeolide cluster in *Streptomyces* sp. NPS554 (data not shown) as well as the synteny of the cluster (Figure 4b). This orthologous cluster present in *L. aerocolonigenes* might be responsible for the production of mangromicins, congeners of akaeolide, isolated from *L. aerocolonigenes* K10-0216 [25]. *Nocardia* sp. BMG51109 is the only organism that has the orthologous genes to the PKS genes for lorneic acids (Table 3) but no secondary metabolites are known from this strain.

### 2.6. Other Type I PKS Gene Clusters in Streptomyces sp. NPS554

**Figure 8:**
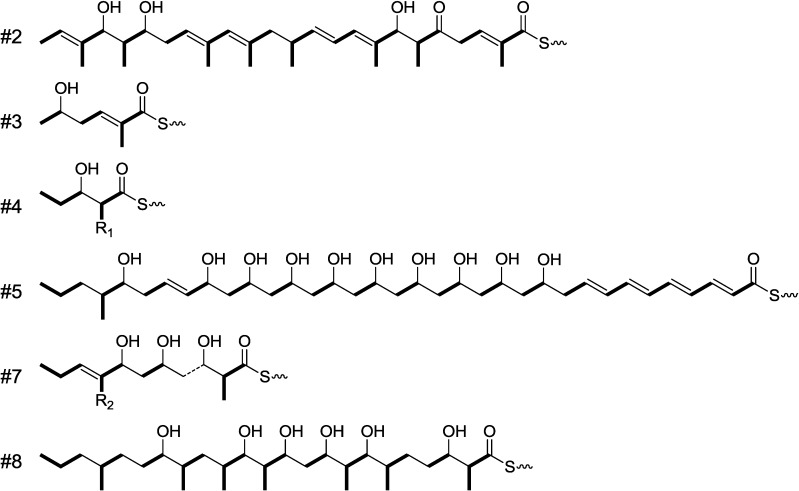
Predicted structures of linear products of orphan type I PKS gene clusters in *Streptomyces* sp. NPS554.

In addition to akaeolide and lorneic acid clusters, eight orphan type I PKS gene clusters are present in *Streptomyces* sp. strain NPS554 (Supplementary Table S1). As for the six clusters completely sequenced, chemical structures of the linear products were predicted on the basis of domain organization (Figure 8) and prediction of stereochemistry of methyl and hydroxyl groups was made (Table S1). Clusters #2 and #8 were predicted to yield linear polyketides comprising of 24- and 23-carbon chain length, respectively, while the products of clusters #3 and #4 were much smaller, consisting of less than ten carbons. The structure of the product from cluster #7 was not completely predicted since one of the PKS modules lacked an AT domain. The side chains R_1_ and R_2_ could not be assigned only from the signature amino acid-sequences. Structures of the products from clusters #9 to #12 could not be predicted because these clusters were not completely sequenced. We recently isolated a new polyhydroxylated tetraene-macrolide corresponding to the product from cluster #5. The detailed structure determination will be reported in our forthcoming paper.

## 3. Materials and Methods

### 3.1. General Experimental Procedures

Sodium [1-^13^C]acetate, [1-^13^C]acetate, sodium [1-^13^C]propionate, and [1,4-^13^C_2_]succinic acid were purchased from Cambridge Isotope Laboratories, Inc. (Andover, MA, USA). ^13^C NMR spectra were obtained on a Bruker AVANCE 400 spectrometer (Bruker BioSpin K.K., Yokohama, Japan). Cosmosil 75C18-PREP (Nakalai Tesque, Inc., Kyoto, Japan, 70 μm) was used for ODS column chromatography. HPLC separation was performed using a Cosmosil 5C18-AR-II (Nacalai Tesque Inc., 20 × 250 mm, Kyoto, Japan) with a photodiode array detector.

### 3.2. Isolation of Akaeolide (**1**) and Lorneic Acid A (**2**)

Strain NPS554 cultured on a Bn-2 slant (soluble starch 0.5%, glucose 0.5%, meat extract (Kyokuto Pharmaceutical Industrial Co., Ltd., Tokyo, Japan) 0.1%, yeast extract (Difco Laboratories, Surrey, United Kingdom) 0.1%, NZ-case (Wako Pure Chemical Industries, Ltd., Osaka, Japan) 0.2%, NaCl 0.2%, CaCO_3_ 0.1%, agar 1.5%) was inoculated into 500 mL K-1 flasks each containing 100 mL of the V-22 seed medium consisting of soluble starch 1%, glucose 0.5%, NZ-case 0.3%, yeast extract 0.2%, Tryptone (Difco Laboratories) 0.5%, K_2_HPO_4_ 0.1%, MgSO_4_·7H_2_O 0.05%, and CaCO_3_ 0.3% (pH 7.0) in natural seawater. The flasks were placed on a rotary shaker (200 rpm) at 30 °C for 4 days. The seed culture (3 mL) was transferred into 500 mL K-1 flasks each containing 100 mL of the A-16 production medium consisting of glucose 2%, Pharmamedia (Traders Protein) 1%, and CaCO_3_ 0.5% in natural seawater. The inoculated flasks were placed on a rotary shaker (200 rpm) at 30 °C for 6 days. At the end of the fermentation period, 100 mL of 1-butanol was added to each flask, which was allowed to shake for 1 h. The mixture was centrifuged at 5000 rpm for 10 min and the organic layer was separated from the aqueous layer containing the mycelium. After evaporation of the solvent, the crude extract was subjected to silica gel column chromatography with a step gradient of CHCl_3_/MeOH (1:0, 20:1, 10:1, 4:1, 2:1, 1:1, and 0:1 v/v). Fractions 2 and 3 (CHCl_3_/MeOH = 20:1 and 10:1) were concentrated to provide a brown solid, which was further purified by repeated reverse phase preparative HPLC using a Cosmosil 5C18-AR-II column (Nacalai Tesque Inc., 10 × 250 mm) with MeCN in 0.1% HCO_2_H (35:65, flow rate 4 mL/min), followed by evaporation and extraction with EtOAc to give akaeolide (**1**, 9.7 mg/L) and lorneic acid A (**2**, 13.5 mg/L).

### 3.3. Chlorination of **1** to Yield 17-Chloroakaeolide (**4**)

To a solution of **1** (5.2 mg, 13.3 μmol) in CH_2_Cl_2_ (1 mL) were added 2,6-lutidine (0.82 μL, 7.1 μmol) and *N*-chlorosuccinimide (3.6 mg, 27 μmol) at room temperature. After stirring for 2 h, the reaction mixture was diluted with diethyl ether (2.5 mL) and washed with brine (1 mL). The organic layer was separated and concentrated *in vacuo* to give 5.8 mg of crude residue. Purification by silica gel chromatography (*n*-hexane/EtOAc=10:1~1:1) yielded 17-chloroakaeolide (**4**, 2.2 mg, 39% yield) as a white powder.

### 3.4. Preparation of ^13^C-Labeled Akaeolide (**1**)

The inoculation, cultivation, extraction, and purification were performed in the same manner as described above. Addition of ^13^C-labeled precursors (1 mL solution/flask) was initiated at 48 h after inoculation and periodically carried out every 24 h for four times. After further incubation for 24 h, the cultures were extracted with 1-butanol.

(1) *Sodium [1-^13^C]acetate*: After feeding of sodium [1-^13^C]acetate (total 800 mg; 20 mg × 10 flasks × 4 days), 9.7 mg of ^13^C-labeled **1** was obtained from 1 L of culture. The ^13^C NMR spectrum showed enriched signals at δ 34.7, 75.4, 140.6, 164.5 and 196.4.

(2) *Sodium [2-^13^C]acetate*: After feeding of sodium [2-^13^C]acetate (total 800 mg; 20 mg × 10 flasks × 4 days), 9.2 mg of ^13^C-labeled **1** was obtained from 1 L of culture. The ^13^C NMR spectrum showed enriched signals at δ 30.1, 43.5, 46.8, 63.7 and 69.0.

(3) *Sodium [1-^13^C]propionate*: After feeding of sodium [1-^13^C]propionate (total 800 mg; 20 mg × 10 flasks × 4 days), 8.7 mg of ^13^C-labeled **1** was obtained from 1 L of culture. The ^13^C NMR spectrum showed enriched signals at δ 28.8, 49.1, 84.1 and 203.1.

### 3.5. Preparation of ^13^C-Labeled Lorneic Acid A (**2**)

The inoculation, cultivation, extraction, and purification were performed in the same manner as described above. Addition of ^13^C-labeled precursors (1 mL solution/flask) was initiated at 48 h after inoculation and periodically carried out every 24 h for 4 times. After further incubation for 24 h the cultures were extracted with 1-butanol.

(1) *Sodium [1-^13^C]acetate*: After feeding of sodium [1-^13^C]acetate (total 800 mg; 20 mg × 10 flasks × 4 days), 13.5 mg of ^13^C-labeled **2** was obtained from 1 L of culture. The ^13^C NMR spectrum showed enriched signals at δ 22.3, 33.0, 121.8, 127.0, 127.4, 131.7 and 177.7.

(2) *Sodium [1-^13^C]propionate*: After feeding of sodium [1-^13^C]propionate (total 800 mg; 20 mg × 10 flasks × 4 days), 8.5 mg of ^13^C-labeled **2** was obtained from 1 L of culture. The ^13^C NMR spectrum showed enriched signals at δ 127.8.

### 3.6. Whole Genome Shotgun Sequencing

Whole genomes of *Streptomyces* sp. NPS554 (=NBRC 109706) and *L. aerocolonigenes* NBRC 13195^T^ were read using a combined strategy of shotgun sequencing with GS FLX+ (Roche; 58 Mb sequences and 7.4-fold coverage for NPS554, 96 Mb sequences and 9.0-fold coverage for NBRC 13195) and paired-end sequencing with MiSeq (Illumina; 705 Mb sequences and 91-fold coverage for NPS554, 770 Mb sequences and 71-fold coverage for NBRC 13195). These reads were assembled using a Newbler v2.6 software, and subsequently finished using a GenoFinisher software [26], which led to a final assembly of 26 and 55 scaffold sequences of >500 bp each for strains NPS554 and NBRC 13195, respectively. The total size of the NPS554 assembly was 7,736,999 bp, with a G+C content of 71.7%, while that of NBRC 13195 one was 10,698,154 bp, with a G+C content of 68.9%. The draft genome sequences of *Streptomyces* sp. NPS554 and *L. aerocolonigenes* NBRC 13195^T^ were deposited in GenBank/ENA/DDBJ under the accession numbers BBOM00000000 and BBOJ00000000, respectively.

### 3.7. Annotation of PKS Gene Clusters

The resulting scaffold sequences were submitted to the in-house auto-annotation pipeline. Coding sequences (CDSs) were predicted by Prodigal v2.6 [27] and searched for domains related to polyketide synthase (PKS) genes such as ketosynthase (KS) domain using the SMART and PFAM domain databases. PKS gene clusters and their domain organizations were determined as reported [28]. Gene functions were assigned according to BLAST searches conducted using the NCBI BlastP program [24] against the non-redundant protein sequences (nr) database. Substrates of each acyltransferase (AT) domains were predicted based on substrate-specific signature amino-acid sequences [12,13].

## 4. Conclusions

Feeding experiments with ^13^C-labeled precursors elucidated the carbon framework construction of akaeolide (**1**) and lorneic acid A (**2**) by PKS pathway. Draft genome sequencing of the producing strain, *Streptomyces* sp. NPS554, and annotation of the gene functions allowed identification of PKS gene clusters for these type I polyketides and their possible biosynthetic pathways that possibly involve uncommon carbon–carbon bond forming reactions. Future investigation will be directed toward the biochemical analysis of the enzymatic reactions. The genome sequence data also allowed mining of orphan type I PKS gene clusters responsible for the production of unknown polyketides. All these results indicate the high potential and uniqueness of secondary metabolism in marine actinomycetes, specifically the group of *Streptomyces*.

## Supplementary Files

Supplementary File 1
