# Supplementary material for: Biosynthesis of Akaeolide and Lorneic Acids and Annotation of Type I Polyketide Synthase Gene Clusters in the Genome of Streptomyces sp. NPS554
Source: Mar Drugs. 2015 Jan 16;13(1):581–96. doi: 10.3390/md13010581 (PMC4306953; doi:10.3390/md13010581)
Supplement: Supplementary File 1 [file marinedrugs-13-00581-s001.pdf]

Supplementary Information

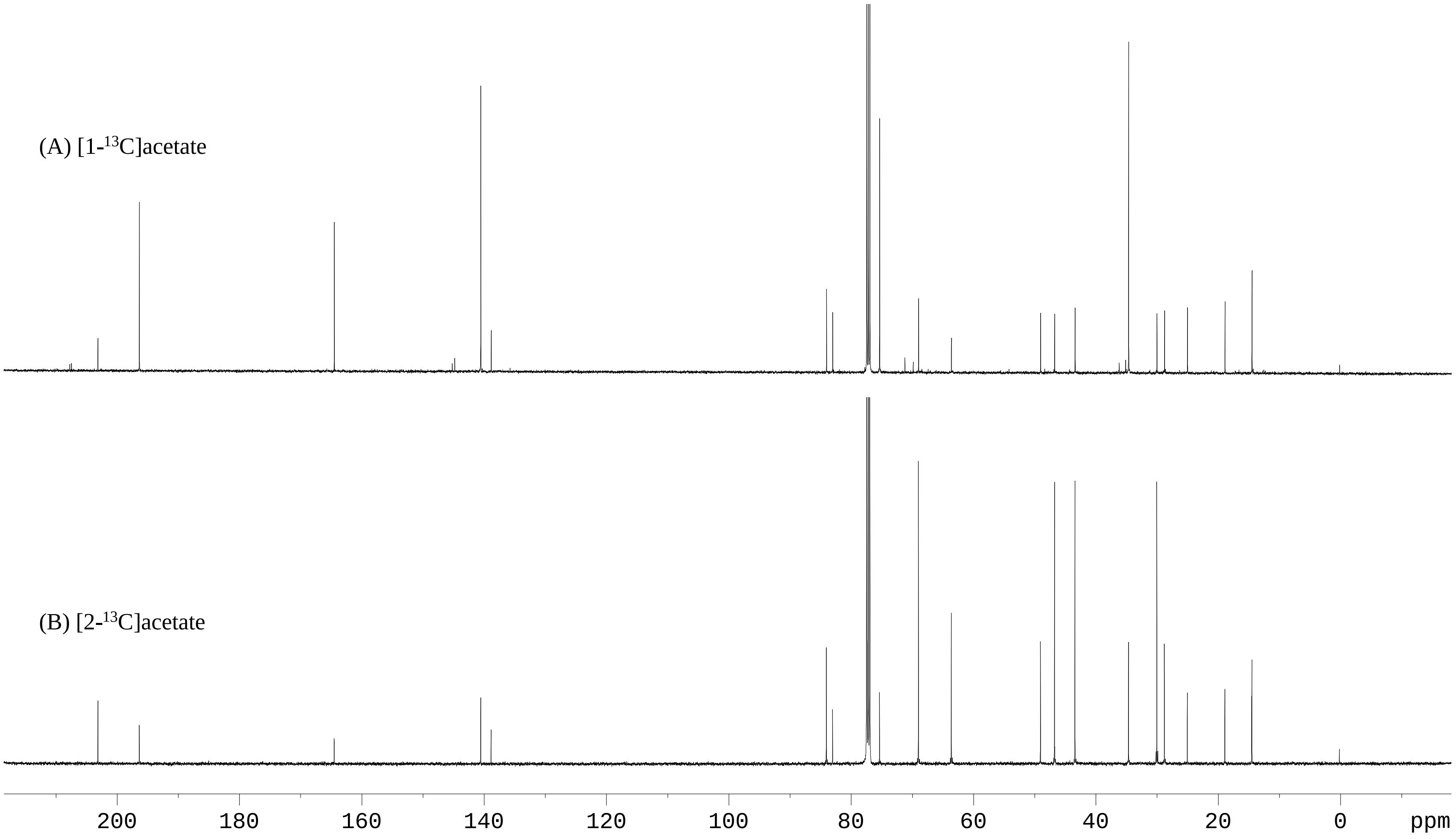

Figure S1. ^{13}C NMR spectra of 17-chloroakaeolide (**4**) labeled with [1-^{13}C]acetate (**A**) and [2-^{13}C]acetate (**B**) (100 MHz, $CDCl_3$).

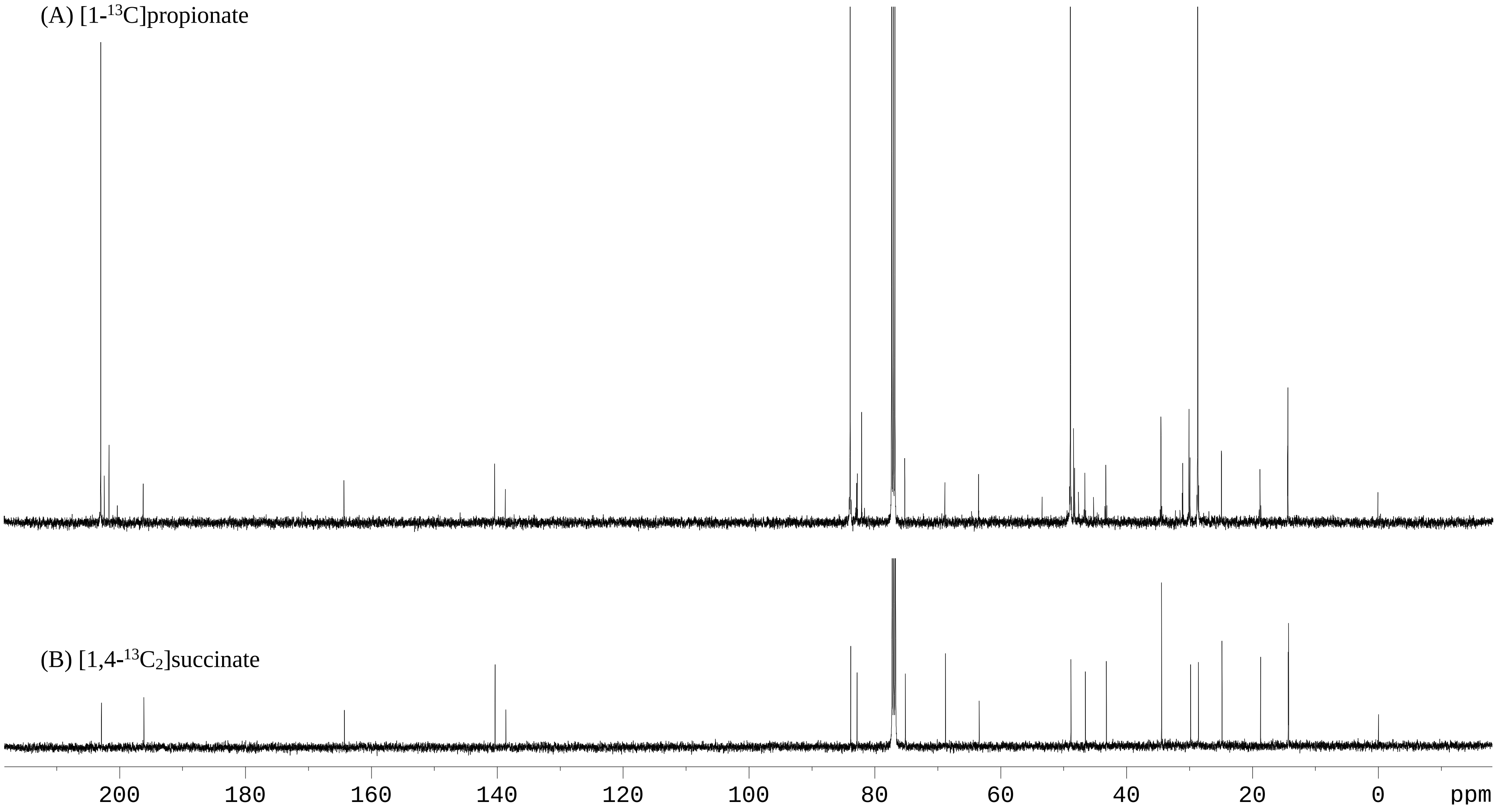

Figure S2. ^{13}C NMR spectra of 17-chloroakaeolide (**4**) labeled with [1-^{13}C]propionate (**A**) and [1,4-$^{13}C_2$]succinate (**B**) (100 MHz, $CDCl_3$).

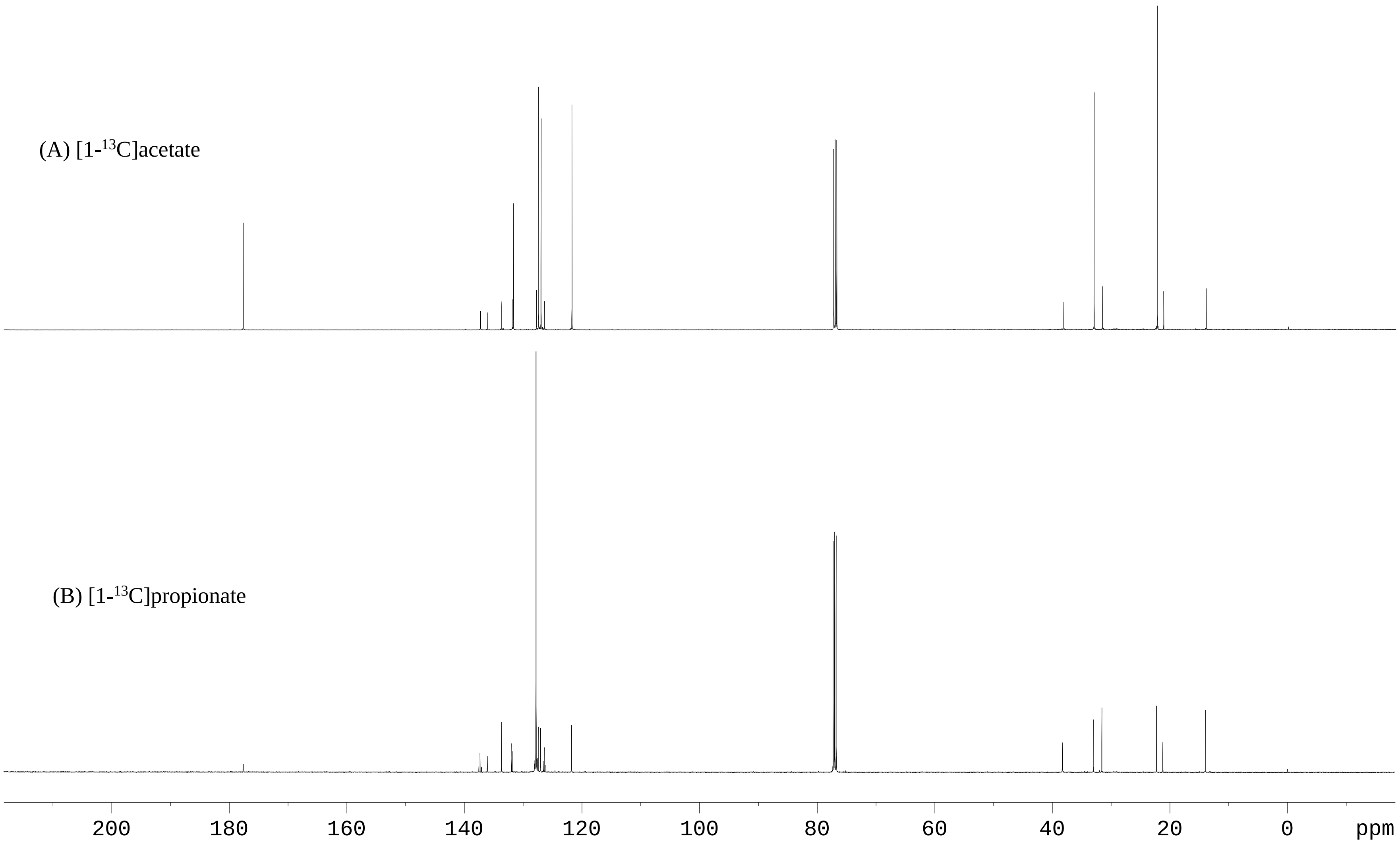

Figure S3. ^{13}C NMR spectra of lorneic acid A (**2**) labeled with [1-^{13}C]acetate (**A**) and [1-^{13}C]propionate (**B**) (100 MHz, $CDCl_3$).

Table S1. Multimodular type I PKS gene clusters in *Streptomyces* sp. NPS554 genome.

Cluster	Module	Orf **	Domain Organization ***
#1	8	1-253	KS/AT/ACP-KS/AT/DH/ER_{S}/KR_{B1}/ACP
		1-254	KS/AT/DH/ER_{S}/KR_{B1}/ACP-KS/AT/DH/KR_{B1}/ACP
		1-255	KS/AT/DH/ER_{S}/KR_{B1}/ACP
		1-256	KS/AT/DH/KR_{B1}/ACP-KS/AT/DH/KR_{B1}/ACP-KS/AT/DH/KR_{B1}/ACP/TE
#2	12	4-545	KS/AT/ACP-KS/ATm/DH/KR_{B1}/ACP-KS/ATm/KR_{B2}/ACP-KS/AT/KR_{A1}/ACP-KS/ATm/DH/KR_{B1}/ACP
		4-544	KS/ATm/DH/KR_{B1}/ACP-KS/ATm/DH/ER_{R}/KR_{B1}/ACP-KS/AT/DH/KR_{B1}/ACP
		4-543	KS/ATm/DH/KR_{B1}/ACP-KS/ATm/KR_{B1}/ACP
		4-542	KS/AT/ACP-KS/ATm/DH/KR_{B1}/ACP/TE
#3	3	5-51	KS/AT/ACP-KS/AT/KR_{B1}/ACP
		5-52	KS/ATm/DH/KR_{B1}/ACP/TE
#4	2	5-363	KS/ATm/ACP-$KR_{?}$
		5-364	KS/ATx
		5-365	ACP
#5	17	8-406	KS/ATm/ACP-KS/ATm/DH/ER_{R}/KR_{B1}/ACP-KS/AT/DH/KR_{B1}/ACP
		8-407	KS/AT/DH/KR_{B1}/ACP-KS/AT/DH/$KR_{?}$/ACP-KS/AT/$KR_{?}$/ACP-KS/AT/KR_{A1}/ACP
		8-408	KS/AT/$KR_{?}$/ACP-KS/AT/KR_{A1}/ACP-KS/AT/KR_{A1}/ACP-KS/AT/KR_{A1}/ACP-KS/AT/KR_{A1}/ACP-KS/AT/DH/KR_{B1}/ACP
		8-409	KS/AT/DH/KR_{B1}/ACP-KS/AT/DH/KR_{B1}/ACP-KS/AT/DH/KR_{B1}/ACP-KS/AT/DH/KR_{B1}/ACP/TE
#6	8	8-520	KS/AT/ACP-KS/ATm/DH/KR_{B1}/ACP-KS/ATm/DH/ER_{S}/KR_{B1}/ACP
		8-521	KS/AT/DH/KR_{B1}/ACP-KS/ATm/DH/KR_{B1}/ACP
		8-522	KS/AT/ACP-KS/ATp/DH/KR_{B1}/ACP
		8-523	KS/AT/ACP/TE
#7	5	9-219	KS/ATm/ACP-KS/ATx/DH/KR_{B1}/ACP
		9-220	KS/AT/$KR_{?}$/ACP
		9-221	KS/$KR_{?}$/ACP-KS/ATm/$KR_{?}$/ACP

Table S1. *Cont.*

#8	9	13-34	KS/ATm/ACP-KS/ATm/DH/ER_{R}/KR_{B1}/ACP
		13-31	KS/AT/DH/ER_{S}/KR_{B1}/ACP
		13-30	KS/ATm/KR_{A2}/ACP
		13-29	KS/ATm/DH/ER_{S}/KR_{B1}/ACP-KS/ATm/KR_{A1}/ACP-KS/AT/KR_{A1}/ACP
		13-28	KS/ATm/KR_{B2}/ACP
		13-27	KS/ATm/KR_{B1}/ACP-KS/AT/DH/ER_{R}/KR_{B1}/ACP
		13-25	KS/ATm/KR_{B2}/ACP-KS
#9 *****	>13	5-1	KS/AT/DH/ER/KR/ACP-KS/AT/KR/ACP-KS/ATm/KR/ACP-KS/AT/DH/KR/ACP
		5-2	KS/AT/DH/KR/ACP-KS/AT/DH/KR/ACP-KS/AT/DH/ER/KR/ACP-KS/AT/DH/ER/KR/ACP KS/AT/DH/KR/ACP
		5-3	KS/AT/KR/ACP KS/ATx/DH/ACP
		5-4	KS/ATm/DH/ER/KR/ACP
		5-5	KS/ATm/DH/ER/KR/ACP/TE
#10 *****	>17	5-412	ACP-KS/AT/KR/ACPKS/AT/KR/ACP-KS/AT/DH/KR/ACP KS/AT/DH/KR/ACP
		5-411	KS/AT/KR/ACP-KS/ATm/KR/ACP-KS/AT/KR/ACP-KS/AT/KR/ACP/TE
		5-409	KS/AT/DH/ER/KR/ACP-KS/ATx/KR/ACP-KS/ATm/DH/KR/ACP
		5-408	KS/AT/KR/ACP
		5-407	KS/AT/KR/ACP-KS/AT/KR/ACP-KS/AT/KR/ACP-KS/AT/DH/KR/ACP-KS/AT/DH/KR/ACP
#11 *****	>3	13-1	KS/AT/ACP-KS/AT/DH/ER/KR/ACP-KS/AT/DH/KR/ACP-
#12 *****	>19	14-1	ATm/DH/KR/ACP-KS/ATm/KR/ACP-KS/AT/DH/ER/KR/ACP-KS/ATm/DH/KR/ACP-KS/AT/KR/ACP-KS/AT/KR/ACP
		14-2	KS/ATm/DH/ER/KR/ACP-KS/ATm/KR/ACP-KS/AT/KR/ACP-KS/AT/KR/ACP-KS/ATm/DH/KR/ACP-KS/AT/KR/ACP
		14-3	KS/AT/KR/ACP-KS/AT/DH/KR/ACP-KS/AT/DH/KR/ACP-KS/AT/DH/KR/ACP-KS/AT/DH/KR/ACP-KS/AT/KR/ACP-KS/ AT/KR/ACP

PKS gene clusters only with a single PKS module and short PKS gene fragments are not included. ***** #9 to #12, partial, probably divided into multiple scaffolds; might be joined to form one cluster. ****** scaffold numbers are shown before hyphen in the orf numbers. KS, ketosynthase domain; AT, acyltransferase domain for malonyl-CoA; ATm, AT for methylmalonyl-CoA; ATp, AT for propylmalonyl-CoA; ATx, AT whose substrate is unpredictable; DH, dehydratase domain; ER, enoylreductatse domain; KR, ketoreductase domain; ACP, acyl carrier protein domain; TE, thioesterase domain. ******* KR is classified into A1-, A2-, B1-, B2-, C1-, C2-type, or unknown (?) based on KR fingerprints [16]. ER is classified into *R*- or *S*-type [17].
